# Adverse Drug Reactions to SGLT2i Reported by Type 2 Diabetes New Users: An Active Surveillance Study

**DOI:** 10.3390/ph18060904

**Published:** 2025-06-16

**Authors:** Camelia Bucșa, Ioana Frenț, Ramona Stefan, Adriana Fodor, Georgeta Inceu, Andreea Farcaș, Adriana Rusu, Monica Negovan, Cristina Mogoșan

**Affiliations:** 1 Pharmacovigilance Research Center, Faculty of Pharmacy, “Iuliu Hatieganu” University of Medicine and Pharmacy, 400349 Cluj-Napoca, Romania; cfarah@umfcluj.ro (C.B.); ioanafrent1@gmail.com (I.F.); afarcas@umfcluj.ro (A.F.); nmonica22@yahoo.com (M.N.); 2 Marymed Medical Center, 420120 Bistrița, Romania; ramonamaria_stefan@yahoo.com; 3 Department of Diabetes and Nutrition Diseases, “Iuliu Hatieganu” University of Medicine and Pharmacy, 400006 Cluj-Napoca, Romania; adriana.fodor@umfcluj.ro (A.F.); georgetainceu@yahoo.com (G.I.); 4 Department of Diabetes, Emergency Clinical County Hospital Cluj, 400006 Cluj-Napoca, Romania; 5 Department 2, Faculty of Nursing and Health Sciences, “Iuliu Hatieganu” University of Medicine and Pharmacy, 400337 Cluj-Napoca, Romania; 6 Directorate of Drug and Medical Device Policy, Ministry of Health, 011478 Bucharest, Romania; 7 Department of Pharmacology, Physiology and Pathophysiology, “Iuliu Hațieganu” University of Medicine and Pharmacy, 400349 Cluj-Napoca, Romania; cmogosan@umfcluj.ro

**Keywords:** safety, patient-reported outcomes, effectiveness, real-world data

## Abstract

**Background/Objectives**: Patients’ perspectives on adverse drug reactions (ADRs) may be used to update the safety profile of a drug. We aimed to prospectively follow-up on type 2 diabetes (T2D) patients who were new users of sodium-glucose co-transporter 2 inhibitors (SGLT2i) and to characterize the patient-reported ADRs within routine practice in Romania. **Methods**: T2D patients from ambulatory settings were interviewed over the phone based on standardized forms, at four time-points across 12 months. We captured the patients’ history and auto-medication, as well as any ADR that implied causality to SGLT2i, based on the patient’s perspective. **Results**: In total, 64 patients, with genders being equally represented and with a median age of 59 years (Q1, Q3: 51, 64) were followed-up with. We identified 73 ADRs to SGLT2i that were suspected to be associated with the drug, with an average of 2.35 ADRs per patient (range 0–7 ADRs/patient). The most reported ADR was pollakiuria (7; 9.58%), followed by vulvovaginal candidiasis (6; 8.21%), dysuria (4; 5.47%), and hypoglycemia (4; 5.47%). SGLT2i treatment was interrupted for eight patients. Three (4.10%) ADRs were considered serious as important medical events (hypertensive crisis, angina pectoris, and dyspnea). A positive dechallenge was recorded for 14 ADRs, of which 9 ADRs had a positive rechallenge as well. A probable causality was assessed for 13 of the 73 patient-reported ADRs. **Conclusions**: Most of the identified ADRs were in line with the known safety profile of SGLT2i. Only three ADRs were serious and unexpected relative to the safety profile, but these had confounding factors that could explain the reactions. Therefore, no new safety concerns related to SGLT2i were determined in this observational study.

## 1. Introduction

Sodium-glucose co-transporter 2 inhibitors (SGLT2i) have an antihyperglycemic effect by reducing the renal reabsorption of glucose, followed by its urinary excretion [1]. In addition to their capacity to lower plasma glucose, they have been shown to reduce weight and blood pressure (BP). Their pleiotropic effects have been proven to bring significant metabolic, renal, and cardiovascular benefits [2,3]. Consequently, dapagliflozin and empagliflozin were approved in the context of two new indications, heart failure (HF) and chronic kidney disease (CKD) [4,5].

Aside from their efficacy, clinical trials have identified several side effects of gliflozins, such as genitourinary infections, hypoglycemia, volume depletion, renal impairment, ketoacidosis, constipation, hyperhidrosis, back pain, dyslipidemia, skin reactions, and increases in creatinine and hematocrit [6,7]. As the use of this class of drugs has increased, additional safety concerns have been identified in the post-marketing phase. Investigations at the level of competent authorities (e.g., European Medicines Agency, United States Food and Drug Administration) have revealed additional risks, such as euglycemic ketoacidosis (with blood glucose level below the threshold for diabetic ketoacidosis), lower limb toe amputations, and necrotizing fasciitis of the perineum. However, investigations concluded that the benefits of these drugs still outweigh their risks [8,9,10,11,12]. Current T2D guidelines recommend SGLT2i use for cardiorenal risk reduction in high-risk patients with T2D [13]. Additionally, SGLT2i utilization has expanded beyond the area of diabetes (heart failure treatment and chronic kidney disease) [4,5], and their increasing use necessitates a closer characterization of their associated adverse drug reactions (ADRs).

Several prospective studies investigated the safety of gliflozins in a real-world context. A comprehensive review of 37 prospective observational studies concluded that while there may be an associated benefit in primary and secondary prevention of cardiovascular disease events, particularly those related to HF, SGLT2i exposure may be associated with an increased risk of genital mycotic infection, lower limb amputation, and euglycemic ketoacidosis. No clear relationships were found for other safety events associated with their use (e.g., fractures, hypoglycemia, and urinary tract infection) [14].

Specific characteristics of the population and healthcare system may affect the use of medication. However, real-world data on Romanian patients are scarce. SGLT2i were investigated in a recent retrospective study on Romanian patients with T2D and found to have beneficial effects on modifiable cardiovascular risk factors [15], but ADRs were not addressed. Another study, which prospectively followed 77 patients with T2D on SGLT2i treatment for 3 months, found that the metabolic and extra-glycemic effects of oral SGLT2i are consistent with those observed in international studies [16]. However, both studies monitored the patients within the context of their usual care. Nevertheless, some of the SGLT2i adverse reactions are not easily recognized by the patients, and others, like urogenital reactions, are sensitive to report during the short duration of the consultation. The active surveillance studies focusing on patient-reported outcomes capture patients’ perspectives and bring more detailed information on the ADRs.

We aimed to investigate SGLT2i effects as reported by the patients in follow-up interviews. The purpose of our study was to follow-up with T2D patients who were new users of SGLT2i to characterize the ADRs and to record the effectiveness parameters available within routine practice in Romania.

## 2. Results

### 2.1. Demographic Characteristics

A total of sixty-seven patients were contacted for this study, of which sixty-four patients were included (one patient did not use the dapagliflozin prescribed by the specialist, and two patients were unreachable at the phone numbers provided for the interviews).

The median age of the population was 59 years (Q1, Q3: 51, 64), and genders were equally represented, with 32 females (50%) and males, respectively. Dapagliflozin was the most used SGLT2i, and the most common concomitant medication was metformin. The most commonly associated diseases were cardiovascular and thyroid disorders (Table 1).

### 2.2. Adverse Drug Reactions

Of the 64 patients included in the study, 31 (48.43%) reported ADRs to SGLT2i. We identified 73 ADRs (T1–T4, Table 2) to gliflozins, as suspected to be associated with the drug, with an average of 2.35 ADRs per patient (range 0–7 ADRs/patient).

Most of the ADRs reported were part of the system organ class (SOC)—Reproductive system and breast disorders (11; 15.06%) and SOC—Renal and urinary disorders (11; 15.06%), followed by SOC—Infections and infestations (8; 10.95%), SOC—Gastrointestinal disorders (7; 9.59%), and SOC—Musculoskeletal and connective tissue disorders (5; 6.84%). The most reported ADR was pollakiuria (7; 9.58%), followed by vulvovaginal candidiasis (6; 8.21%), dysuria (4; 5.47%), and hypoglycemia (4; 5.47%) (Supplementary Table S1). SGLT2i treatment was interrupted in eight patients (five males) due to fifteen ADRs, six of which were genitourinary. Of the eight patients, four stopped the treatment with SGLT2i after less than 1 month, one patient stopped the treatment after 3 months, two patients stopped after 5 months, and one patient stopped after 9 months of SGLT2i utilization.

The majority of ADRs (70; 95.89%) were non-serious. Three (3; 4.10%) ADRs were considered serious, and the seriousness criteria described an important medical event in all three cases. The serious ADRs reported were hypertensive crisis (1), angina pectoris (1), and dyspnea (1).

Of the 73 SGLT2i patient-reported ADRs, fourteen had a positive dechallenge and nine had a positive dechallenge and rechallenge. The nine ADRs with a positive dechallenge and rechallenge (headache, pollakiuria, genital rash, genital erosion, genital pain, pruritus genital, penile blister, penile exfoliation, and penile erythema), together with four of the ADRs with positive dechallenge (e.g., genital erosion, cheilitis, increased blood glucose, and vulvovaginal candidiasis) were assessed as having a probable causality (Table 2). The patient who reported an increase in blood glucose also reported that she had interrupted the utilization of dapagliflozin due to this event after 1 month of use. However, the attending diabetologist did not confirm this ADR, as her HbA1c had slightly decreased at the 3-month follow-up visit (−0.58%).

The majority (48, 65.75%) of the ADRs reported in the study are in line with the known safety profile of the SGLT2i. Twenty-five (34.25%) of the patient-reported ADRs were unexpected, when compared with the European SPCs of the SGLT2i as of 2024 (Supplementary Table S1). However, of the 25 unexpected ADRs, 22 (88.88%) were non-serious. This leads to the conclusion that three ADRs were both serious and unexpected, given the safety profile of SGLT2i. These three ADRs also presented confounding factors (underlying disease) and therefore demonstrated only a “possible” causal relation with dapagliflozin.

We also performed an exploratory logistic regression analysis aiming to investigate baseline patient characteristics associated with ADR occurrence. Neither sex nor age, nor diabetes chronic complications (chronic kidney disease, retinopathy, and peripheral neuropathy), nor arterial hypertension, nor cardiovascular diseases (ischemic heart disease, stroke, and congestive heart failure) explained the occurrence of the ADRs in our sample (*p* > 0.1 for all).

### 2.3. Effectiveness

Most of the measurements for effectiveness were available at the start (T0) and at 3 months after the commencement of use of SGLT2i (T1). The median HbA1c decreased significantly 3 months after the use SGLT2i, from 8% to 7% (T1 vs. T0 *p* = 0.001), and further, to 6.80%, after another 3 months of SGLT2i use (T2 vs. T0 *p* = 0.028). The lowest median HbA1c was observed at 9 months after the start of the SGLT2i (6.65%), but the value at this time point was obtained only for 6 patients. The median fasting blood glucose was lowest at 6 months after the SGLT2i start (T2) (Table 3).

The median weight loss was highest at one year after the start of SGLT2i (4.5 kgs; Q1, Q3: 3.25–5.75). Three of the sixty-four patients included in our study gained weight, compared to their initial status (Table 3).

The median creatinine remained stable during the one year of follow-up for all patients with available measurements (Table 3).

## 3. Discussion

This study aimed to closely follow-up with patients for 12 months after the start of their treatment with SGLT2i. We included in our follow-up 64 T2D patients, and about half of them (48.43%) reported at least one ADR related to SGLT2i. The percentage of patients with ADRs is higher than the 15% reported by Unadkat et al. in a prospective study of Indian patients [17] and also higher than that reported by Hopf et al. in a retrospective study of German patients (26.3%), as well as that reported by Mirabelli et al. in a retrospective study of Italian patients (25%) [18,19]. One possible explanation is the method of intensive monitoring of our study, in which the researchers spent more time with the patient and documented more details, in addition to the fact that we included all ADRs that patients reported to be due to SGLT2i independent of medical confirmation of the event. Also, the disparity might have arisen from the careful selection of patients by Unadkat et al. [17] and the different definitions of the ADRs in the retrospective studies of patients who stopped the treatment with SGLT2i due to ADRs [18,19]. Our study was the first prospective active surveillance performed in Romanian patients who started SGLT2i according to the local prescribing practice in place, as regulated by the National Health Insurance Company.

The ADRs identified in this study are largely the ones well known from other real-world studies [14,18,19,20,21,22,23,24] and those already listed in the products’ information. Genital mycotic infections and a borderline increase in urinary tract infections are the most common reported ADRs with SGLT2i. However, more serious life-threatening ADRs, such as euglycemic ketoacidosis, amputations, and Fournier gangrene, have also been reported in a small number of users, generating warnings from regulatory agencies [21,22]. In our sample, the genitourinary ADRs, known to be the most frequent concerns in SGLT2i users, represented almost 40% of the total number of ADRs, which is in line with the SGLT2i safety profile. Of these, only genital erosion (one patient, possible causality) was not listed in the empagliflozin product information [5]. No rare ADRs were reported in our sample. This does not mean that they do not occur, as reports of euglycemic ketoacidosis have also emerged from Romanian users of SGLT2i [25].

Other unexpected ADRs, either with (18% of all ADRs) or without (19% of all ADRs) confounding factors, had a weak causal relation to the SGLT2i, and so were probably coincidental events. The only unexpected ADR with a probable causality was the incidence of headache, with no known alternative explanation for the patient, and with positive dechallenge and rechallenge. The headache appeared 2 days after the start of dapagliflozin, resolved without treatment after dapagliflozin withdrawal, and reappeared when dapagliflozin was restarted. According to the patient (54, M), his hydration was appropriate, and his blood pressure did not reach low levels. Headache associated with SGLT2i was previously reported in a multicenter retrospective descriptive analysis evaluating Kaiser Permanente Southern California members with type 2 diabetes and chronic kidney disease 1, 2, or 3 who first filled an SGLT2 inhibitor prescription [26]. Although it has been described as a symptom of probable euglycemic ketoacidosis, the exact mechanism by which SGLT2 inhibitors induce headaches is unknown [27]. SGLT2 receptors have been identified in the central nervous system, and a positive effect of SGLT2 inhibitors in neurological disorders has been hypothesized based on the effects of these drugs on neuron membrane potential through the modulation of sodium transport [28,29]. Thus, we can speculate that the presence of SGLT2 receptors in the nervous system may explain the headache reported in our sample.

Of the total of 73 patient-reported ADRs, three were serious, comprising one patient with hypertensive crisis, and another patient with angina pectoris and dyspnea, none of them medically confirmed. The hypertensive crisis (200/100 mmHg, important medical event, ER visit) appeared approximately one month after the initiation of the dapagliflozin treatment. The patient (56-year-old female) had a history of hypertension and ischemic stroke. The hypertensive crisis is not a known adverse reaction of dapagliflozin; on the contrary, SGLT2i were shown to reduce blood pressure (BP). Although the mechanisms underlying the BP-lowering effects of SGLT2i are unclear, SGLT2i presumably act primarily by decreasing circulating plasma volume through osmotic and natriuretic diuresis in the early stages of administration and by suppressing sympathetic nerve activity in the long term [30]. The temporal association between dapagliflozin administration and the onset of the event does not exclude a possible causality. However, the underlying disease is a more plausible cause of the event. Besides dapagliflozin, the patient was using metformin, gliclazide, indapamide, bisoprolol, zofenopril, and moxonidine. However, there are no known drug interactions in this patient’s treatment that could lead to hypertension [31].

Angina pectoris and dyspnea occurred in a 43-year-old female patient with a 3-year history of T2D, hypertension, and peripheral edema, who received treatment with dapagliflozin. The patient was also using the following concomitant medications: metformin, insulin glargine, enalapril, and indapamide. Approximately five months after the initiation of dapagliflozin, the patient experienced angina pectoris and dyspnea (which were considered important medical events as per the seriousness criteria [32]). The temporal association between dapagliflozin administration and the onset of the events may be compatible, but the medical history (peripheral edema that occurred a few years ago and hypertension) suggests the onset of atherosclerotic coronary disease and/or heart failure. A review looking at the effects of SGLT2i on ischemic events stemming from atherosclerotic coronary diseases showed that SGLT2i have no significant effect on ischemic events [33].

The effectiveness parameters recorded, namely, HbA1c, weight loss, and fasting blood glucose, were in line with the results from other observational studies. With a median drop of 1% in HbA1c at 6 months, a peak in weight loss at 12 months, and a stable renal function, our results are comparable with the SOLD study [34].

Our study reinforces existing evidence that genitourinary adverse effects are the most frequently reported ADRs associated with SGLT2i use, highlighting the need for proactive patient education on recognizing and managing these reactions. Given that nearly half of the patients reported at least one ADR, our findings suggest that active surveillance using patient-reported outcomes can uncover a higher frequency of ADRs than passive pharmacovigilance methods.

### Strengths and Limitations

The strength of our study was the level of detail that we managed in discussions with patients during the interviews. Patient-reported outcomes, even though they are subjective and the quality and accuracy of collected data is variable, bring valuable insights from the patient’s perspective. We combined the data retrieved from patients’ interviews with data from the medical charts to have a more complete picture of the effects of the SGLT2i. Many observational studies only investigate the rate of treatment interruptions due to urogenital ADRs and with this approach, the opportunity to identify new ADRs is limited. We registered all ADRs that were deemed by the patients to be related to their SGLT2i use, and we performed the causality analysis for each of the ADRs. Another strength of our study was the follow-up of 12 months for each patient and the interviews performed by researchers experienced in pharmacovigilance.

The main limitation of our study is the small group of patients and the fact that we did not have all the data for all the patients at each time point. We captured the data as collected in real-world practices. Another limitation is that we did not have medical confirmations of the patient-reported ADRs, nor did we have a cross-validation from the patients’ reports. However, observational prospective studies performed without funding are still valuable and have other advantages, like patient diversity and the provision of real-world data. Also, we have not assessed the drug interactions which may have been associated with the ADRs observed in our sample.

## 4. Materials and Methods

### 4.1. Study Design

This prospective active surveillance study included patients from the northwest region of Romania.

The study duration was 18 months, extending between 15 March 2019 and 18 September 2020. During this period, patients with a new prescription of SGLT2i were interviewed at 4 time points, at 3 (T1), 6 (T2), 9 (T3), and 12 (T4) months after starting the therapy. Each interview was performed over the telephone, within 10 days after each time point. The time point was considered missed if the patient could not be contacted within these 10 days.

The study was approved by the Ethics Committee of the Iuliu Hatieganu University of Medicine and Pharmacy Cluj-Napoca (No 62/11.03.2019). All participants were informed about the study-related procedures and personal data collection and signed an informed consent form before data collection or any interviews. Data were collected using paper forms especially designed for data from both medical charts and phone interviews; each participant received a study number and data were recorded into a password-protected Microsoft Excel file. Only investigators involved in data collection had access to personal data that allowed for the participants’ identification and follow-up.

### 4.2. Patients

Patients registered at 3 ambulatory diabetologist offices were included in the study if (1) they were over 18 years of age and (2) without any cognitive impairment documented in the patient record, (3) they had started treatment with SGLT2i no more than 3 months prior, and (4) they had signed the informed consent form describing participation in the study. The SGLT2i available in Romania (dapagliflozin and empagliflozin), were considered in any concentrations and combinations (e.g., dapagliflozin 10 mg, dapagliflozin 10 mg + saxagliptin 5 mg, and empagliflozin 10 mg).

The decision to initiate the SGLT2i therapy was taken by the attending diabetologist based on the national prescribing protocols in place at the time. The current study did not affect prescribing and monitoring practice.

### 4.3. Data Collection

We used patients’ charts to extract information on the SGLT2i start date, T2D diagnostic date, comorbidities, co-medication, diabetes complications, laboratory data (HbA1c, fasting glucose, creatinine, urea, hepatic enzymes, and cholesterol), weight or body mass index (BMI), and results of other available examinations. This data was extracted if already available in the patient’s chart, and extra tests or examinations for this study were not required.

Patients’ interviews were performed over the phone, by a physician or a pharmacist with training in pharmacovigilance, using standardized forms. The forms contained open and closed questions on the patient’s history, self-medication, and a specific set of events (ketoacidosis, dehydration, hypoglycemia, hypotension, and urinary/genital, digestive, skin, and feet abnormalities). In addition, we included open questions regarding any other unusual symptoms that might have occurred since the start of the SGLT2i. In case of a reported adverse event, another form was filled in with information on the temporal relation, symptoms, duration, severity, seriousness, treatment, evolution, de-challenge, re-challenge, and outcome. The key question in this case was “Do you feel that this event is connected with the use of your new medication?” Additionally, to ensure the uniformity of the interviews, the first 3 interviews were performed by one investigator in the presence of the other investigators. This study captured only ADRs, meaning adverse events that imply causality, from the patient’s perspective.

### 4.4. Data Analysis

No sample size calculation was performed and a convenience sample of 64 patients was selected.

We applied the WHO-UMC system for standardized case causality assessment to all the adverse events that were perceived as being due to the SGLT2i, from the patient’s perspective [35]. The expectedness of the ADRs was assessed in comparison to the Summary of Product Characteristics (SPC) [4,5].

We performed a descriptive statistical analysis of the data collected. Continuous variables were expressed as median and interquartile range (Q1, Q3), and categorical variables as numbers and percentages.

HbA1C, fasting plasma glucose, weight, and creatinine at first prescription were compared with those at different time points, for each patient with available data using the Related-Samples Wilcoxon Signed Rank test. An exploratory logistic regression with comorbidities, age, and sex at baseline was used to investigate potential predictors of the ADRs. Statistical analysis was performed using Microsoft Excel and SPSS version 26.0 (IBM Corp, Armonk, NY, USA). For all analyses, a *p*-value < 0.05 was considered statistically significant.

## 5. Conclusions

This is the first prospective active surveillance study aiming to assess the safety profile of SGLT2i in Romanian patients with type 2 diabetes, in real-life settings and considering the particularities of the Romanian healthcare system. In our sample, most of the identified ADRs were in line with the known safety profile of SGLT2i. Only three ADRs were serious and unexpected relative to the safety profile, but these had confounding factors. Therefore, no new safety concerns related to SGLT2i were derived from this observational study. Although this study adds to the local knowledge relevant to pharmacovigilance and SGLT2i, larger real-life studies performed in the Romanian population are required in order to completely describe the safety profiles of these drugs.

## Figures and Tables

**Table 1:** Demographic characteristics of patients at the start of treatment with SGLT2i (T0, N = 64).

Patients (N = 64)	
Median Age (Q1, Q3)	59 (51, 64)
Median Age at DM Diagnosis (Q1, Q3)	52 (48, 57)
Gender, n (%)	32 (50.00%)
**Drugs, n (%)**	
Dapagliflozin 10 mg	36 (56.25%)
Dapagliflozin 10 mg + Saxagliptin 5 mg	5 (7.81%)
Empagliflozin 10 mg	23 (35.93%)
**Diabetes Mellitus Complications, n (%)**	
Neuropathy	10 (15.62%)
Retinopathy	8 (12.50%)
Diabetic foot	3 (4.68%)
Nephropathy	1 (1.56%)
**Associated Diseases, n (%)**	
Arterial Hypertension	42 (65.62%)
Cardiovascular Disease	
Ischemic Cardiomyopathy	23 (35.93%)
Heart Failure	6 (9.35%)
Myocardial Infarction	3 (4.68%)
Ischemic Stroke	2 (3.12%)
Peripheral Vascular Diseases	2 (3.12%)
Dyslipidemia	20 (31.20%)
Thyroid Disorder (Hypothyroidism, Multinodular Goiter, Thyroid Surgery)	10 (15.62%)
Obesity	6 (9.35%)
Hyperuricemia, Gout	3 (4.68%)
Hepatic Steatosis	3 (4.68%)
Pancreatitis	2 (3.12%)
Hepatitis (B)	1 (1.56%)
**Concomitant Medication, n (%)**	
Biguanide (Metformin)	48 (75.00%)
Diuretics (Indapamide, Furosemide, Spironolactone)	21 (32.81%)
ACE inhibitors (Fosinopril, Enalapril, Ramipril, Perindopril)	19 (29.69%)
Statins (Atorvastatin, Rosuvastatin, Simvastatin)	16 (25.00%)
Gliclazide	10 (15.63%)
Beta-blockers (Metoprolol, Bisoprolol)	10 (15.63%)
Insulin	14 (21.87%)
Glargine	12
Detemir	1
Glulisine	1
Rapid	5
Insulin (Not Specified)	2
Calcium Channel Blockers (Amlodipine)	5 (7.81%)
Angiotensin Receptor Blockers (Candesartan, Valsartan)	3 (4.69%)
**Tobacco Use, n (%)**	4 (6.25%)
**Alcohol Use, n (%)**	3 (4.69%)

SGLT2i, sodium-glucose cotransporter 2 inhibitors; ACE, angiotensin-converting enzyme; n, number of patients in a given category.

**Table 2:** The characteristics of the ADRs.

Characteristic	Number of ADRs
* Time point	
T1	49
T2	27
T3	5
T4	4
Time to onset	
<1 day	7
1–7 days	7
8–90 days	33
91–180 days	10
181–270 days	3
>270 months	2
Unknown	11
Expectedness	
Expected	46
Unexpected	27
Causality	
Probable	15
Possible	57
Not assessable	1
ADR duration	
<1 day	9
1–7 days	11
8–30 days	17
31–60 days	1
61–90 days	2
>90 days	12
Unknown	21
ADR treatment (yes)	30
Outcome	
Resolved	37
Recovering	13
Ongoing	13
Unknown	10
Confounding factors (yes)	36
Dechallenge (positive)	14
Rechallenge (positive)	10

ADR, adverse drug reaction; *, 12 of the ADRs identified at T1 continued to T2 or T3.

**Table 3:** Effectiveness measurements during the study.

	T0	T1	*p*-Value vs. T0	T2	*p*-Value vs. T0	T3	*p*-Value vs. T0	T4	*p*-Value vs. T0
Median HbA1c (%) (Q1, Q3)	8.00 (7.60–8.56)	7.07 (6.65–7.751)	0.001	6.80 (6.66–7.25)	0.028	6.65 (6.40–7.00)	0.043	7.00 (6.35–7.38)	0.007
Median FBG (mg/dL)	171.00 (144.00–214.5)	149.50 (140.00–159.00)	0.007	148.00 (141.00–164.0)	0.068	151.00 (138.00–153.00)	0.655	120.00 (116.00–144.00)	0.042
Creatinine, mg/dL	0.80 (0.69–0.90)	0.80 (0.73–0.95)	0.969	0.70 (0.69–0.90)	0.042	0.80 (0.79–0.90)	0.655	0.79 (0.77–0.97)	0.317

HbA1c: glycated hemoglobin; FBG: fasting blood glucose; Q1, Q3: 1st and 3rd quartile.

## Data Availability

The original contributions presented in this study are included in the article/Supplementary Materials. Further inquiries can be directed to the corresponding author.

## Supplementary Materials

The following supporting information can be downloaded at: https://www.mdpi.com/article/10.3390/ph18060904/s1, Supplementary Table S1: Summary Tabulations of Adverse Drug Reactions Reported as Being Associated with SGLT2i.

## Abbreviations

The following abbreviations are used in this manuscript:

ADR	Adverse drug reaction
T2D	Type 2 diabetes
SGLT2i	Sodium-glucose co-transporter 2 inhibitors
