# Supplementary material for: Adverse Drug Reactions to SGLT2i Reported by Type 2 Diabetes New Users: An Active Surveillance Study
Source: Pharmaceuticals (Basel). 2025 Jun 16;18(6):904. doi: 10.3390/ph18060904 (PMC12196258; doi:10.3390/ph18060904)
Supplement: Supplementary file 1 [file pharmaceuticals-18-00904-s001.zip › pharmaceuticals-3656644-supplementary.pdf]

Supplementary Table S1. Summary Tabulations of adverse drug reactions reported to SGLT2i

**System Organ Class (SOC)**<br>Preferred Term (PT)	**Serious**	**Non-serious**	***Expectedness**	**Total**
**Cardiac disorders**	**1**	**2**		**3**
Angina pectoris	1	0	Unexpected	1
Palpitations		1	Unexpected	1
Tachycardia		1	Unexpected	1
**Eye disorder**	**0**	**1**		**1**
Visual impairment, condition aggravated		1	Unexpected	1
**Gastrointestinal disorders**	**0**	**7**		**7**
Abdominal discomfort		1	Expected	1
Cheilitis		1	Unexpected	1
Constipation		2	Expected	2
Diarrhea		1	Unexpected	1
Nausea		2	Unexpected	2
**General disorders and administration site conditions**	**0**	**2**		**2**
Oedema peripheral, condition aggravated		1	Unexpected	1
Oedema peripheral		1	Unexpected	1
**Infections and infestations**	**0**	**8**		**8**
Fungal foot infection		1	Unexpected	1
Urinary tract infection		1	Expected	1
Vulvovaginal candidiasis		6	Expected	6
**Investigations**	**0**	**3**		**3**
Blood glucose increased		1	Unexpected	1
Creatinine increased		1	Expected	1
Glycosylated hemoglobin increased		1	Expected	1
**Metabolism and nutrition disorder**	**0**	**4**		**4**
Hypoglycemia		4	Expected	4
**Musculoskeletal and connective tissue disorders**	**0**	**5**		**5**
Back pain		3	Expected	3
Musculoskeletal pain		1	Unexpected	1
Pain in extremity		1	Unexpected	1
**Nervous system disorder**	**0**	**4**		**4**
Dizziness		1	Expected	1
Headache		1	Unexpected	1
Paresthesia, condition aggravated		1	Unexpected	1
Somnolence, condition aggravated		1	Unexpected	1
**Penile and scrotal disorder (excl infections and inflammations)**	**0**	**4**		**4**

Penile blister		1	Expected	1
Penile erythema		2	Expected	2
Penile exfoliation		1	Expected	1
**Psychiatric disorders**	**0**	**1**		**1**
Confusion		1	Unexpected	1
**Renal and urinary disorders**	**0**	**11**		**11**
Dysuria		4	Expected	4
Pollakiuria		7	Expected	7
**Reproductive system and breast disorders**	**0**	**11**		**11**
Genital discomfort		1	Expected	1
Genital erosion		2	Unexpected	2
Genital pain		1	Expected	1
Genital rash		1	Expected	1
Pruritus genital		3	Expected	3
Vulvovaginal disorder		1	Unexpected	1
Vulvovaginal pruritus		2	Expected	2
**Respiratory thoracic and mediastinal disorders**	**1**	**1**		**2**
Cough		1	Unexpected	1
Dyspnea	1	0	Unexpected	1
**Skin and subcutaneous tissue disorder**	**0**	**4**		**4**
Pruritus		1	Expected	1
Rash		1	Expected	1
Rash pruritic		2	Expected	2
**Vascular disorders**	**1**	**2**		**3**
Flushing		1	Unexpected	1
Hypertensive crisis	1	0	Unexpected	1
Hypotension		1	Expected	1
Total PTs	**3**	**70**		**73**
**Cumulative case count**				**31**

SGLT2i, sodium glucose co-transporter 2 inhibitors *, according to the Summary of Product Characteristics of Forxiga® and Jardiance®
